# Study on the Effectiveness of Simultaneous Recovery and Concentration of 1-Ethyl-3-methylimidazolium Chloride Ionic Liquid by Electrodialysis with Heterogeneous Ion-Exchange Membranes

**DOI:** 10.3390/ijms222313014

**Published:** 2021-12-01

**Authors:** Dorota Babilas, Anna Kowalik-Klimczak, Piotr Dydo

**Affiliations:** 1 Department of Inorganic, Analytical Chemistry and Electrochemistry, Faculty of Chemistry, Silesian University of Technology, B. Krzywoustego 6, 44-100 Gliwice, Poland; piotr.dydo@polsl.pl; 2 Bioeconomy and Eco-Innovation Centre, Łukasiewicz Research Network—The Institute for Sustainable Technologies, Pułaskiego 6/10, 26-600 Radom, Poland; anna.kowalik-klimczak@itee.radom.pl

**Keywords:** 1-ethyl-3-methylimidazolium chloride, ionic liquids, ionic liquids recovery, electrodialysis, membrane processes

## Abstract

Due to the extensive range of ionic liquids (ILs) used in industry, an efficient recovery method is needed. In this study, the effectiveness of a simultaneous concentration and recovery method was investigated for 1-ethyl-3-methylimidazolium chloride ([Emim]Cl), an IL that was recovered using electrodialysis (ED). The optimal operational parameters for electrodialytic recovery were determined empirically. The variables that were investigated included the concentration of IL, applied voltage, linear flow velocity and the diluate-to-concentrate volume ratio. The recovery of [Emim]Cl, the concentration degree, the [Emim]Cl flux across membranes, the current efficiency, as well as the energy consumption were determined. The results of the experiments confirmed that [Emim]Cl concentration and recovery can be achieved using ED. The highest ED efficiency was obtained when a 2 V electric potential per one membrane pair was applied, using a 2 cm/s linear flow velocity, and by adjusting to 0.2 M IL in the feed solution. By using ED, a 2.35-fold concentration of [Emim]Cl with a recovery of 90.4% could be achieved when the diluate-to-concentrate volume ratio was 2. On the other hand, a 3.35-fold concentration of [Emim]Cl with a recovery of 81.7% could be obtained when the diluate-to-concentrate volume ratio was increased to 5.

## 1. Introduction

An ionic liquid (IL) is a liquid salt. The melting points of ILs are generally below room temperature, occasionally below 0 °C, and they are classified as molten salts [1,2]. ILs are characterized, for example, by high thermal and chemical stability, negligible vapor pressure, non-flammability and a wide electrochemical window. However, it should be noted that ILs may be toxic [3,4]. Due to their unique chemical and physical properties, they can be used in fields such as in wastewater treatment, electrochemistry, catalysis, analysis and biomass valorization [5]. ILs are also classified on the basis of their properties and their applications, such as task-specific ILs, supported ILs, chiral ILs, protic ILs, bio ILs, polarizable ILs, metal salt ILs, switchable-polarity solvents and deep eutectic solvents [6,7]. ILs are regarded as green solvents because of their recyclability and the potential to replace volatile organic solvents [8,9,10].

ILs are often used for the treatment of environmental samples, such as for the treatment of lignocellulosic biomass. Imidazolium ILs can liquefy lignin, cellulose and therefore wood. A reagent that is commonly used as a solvent for the liquefaction of wood is 1-ethyl-3-methylimidazolium chloride ([Emim]Cl). [Emim]Cl allows lignin and polysaccharides to be liquefied at temperatures around 100 °C. Thus, [Emim]Cl can be used as an alternative solvent in cellulose derivatization reactions, instead of the conventional organic solvents that are characterized by toxicity and volatility [11,12,13].

However, ILs are expensive and during biomass treatment they are diluted to a low concentration. The 1,3-alkylimidazolium ILs are often used to dissolve and regenerate cellulose. Cellulose regeneration consists of a precipitation stage with a water addition, in which a large volume of dilute IL wastewater is produced. The IL concentration in wastewater from biomass utilization is generally in the range from 0.04 to 0.25 M [14,15]. Moreover, ILs can be hazardous to the environment if they are not adequately contained or treated. Wastewater containing ILs can be toxic to microorganisms, people and the environment, especially aquatic or marine ecosystems [13,16,17]. Therefore, it is very important to develop methods to concentrate and recover ILs from waste solutions.

Reclamation technology for ILs remains complex and poorly explored. Due to the different natures of wastewater containing ILs, each will require a unique recovery method. The recovery methods that are used or under development include distillation, molecular distillation, melt crystallization, liquid–liquid separation, absorption and membrane separation [18]. Membrane separation is explored further in the present study.

One of the membrane techniques that has demonstrated potential for the recovery of ILs from aqueous solutions is electrodialysis (ED) [19,20]. ED is a process in which ions from dissolved salts migrate across electrically charged ion-exchange membranes by applying an external electric field as the driving force. The ED stack consists of alternate cation- and anion-exchange membranes between two electrodes. In the ED stack, cations migrate through cation-exchange membranes toward the cathode, and negatively charged anions migrate through anion-exchange membranes toward the anode, and consequently they are retained by the anion- and cation-exchange membranes, respectively. Therefore, in the ED process, the concentrated and diluted solutions are produced [20,21,22].

In the published literature, several studies describe the recovery of ILs using the ED method. It is clear that ED is a favored method, but its effectiveness depends on the operating parameters. The published literature is focused on the recovery of imidazolium ILs, especially 1-allyl-3-methylimidazolium chloride ([Amim]Cl) [14], 1-butyl-3-methylimidazolium bromide ([Bmim]Br) [23], 1-butyl-3-methylimidazolium chloride ([Bmim]Cl) [15,24], 1-butyl-3-methylimidazolium hydrogensulfate ([Bmim]HSO_4_) [25,26] and triethylammonium hydrogen sulfate [TEA][HSO_4_] [27]. It has been demonstrated that the efficiency of the method depends on the type of IL. It was proved that, depending on the examined ILs, ED can be an efficient and quick method for IL recovery from aqueous solution, allowing for IL recovery with a recovery ratio ranging from 50 to 96%. The effectiveness of recovery also depends on the arrangement of the ion-exchange membranes in the ED module and the composition of the electrode rinse solution. It was noted that when the ILs solution was also applied as the electrode rinse solution, the recovery of ILs using ED increased in efficiency [26].

ILs can be recovered by bipolar membrane electrodialysis or integrated membrane systems [23,25,27]. Moreover, there is a consensus in the published literature that the application of ED for the recovery of ILs should continue to be investigated and improved. It was noted that the recovery of ILs is highly dependent on ED parameters such as the initial concentration, voltage, linear flow velocity and diluate-to-concentrate volume ratio. Therefore, it is important to select the optimal ED parameters for each specific IL.

The aim of this work is to investigate the effectiveness of simultaneous recovery and concentration of [Emim]Cl in a laboratory-scale ED module. The influence of the ED parameters on the effectiveness of [Emim]Cl concentration and recovery is discussed in detail. The successful recovery of [Emim] by ED has not yet been demonstrated in the available literature; therefore, it could be a novel way to recover and recycle [Emim]Cl from wastewater.

## 2. Materials and Methods

### 2.1. Experimental Solutions

The experiments were conducted using the configuration defined in Section 2.2, with a solution containing aqueous [Emim]Cl (Sigma Aldrich, Saint Louis, MO, USA). A sulfuric acid solution with a conductivity of 20 mS/cm was used as the electrode rinse solution. The solution compositions are presented in Table 1. Deionized water was prepared using a Millipore Elix 10 system.

### 2.2. ED Stack, Membranes and ED Method

**Figure 1:**
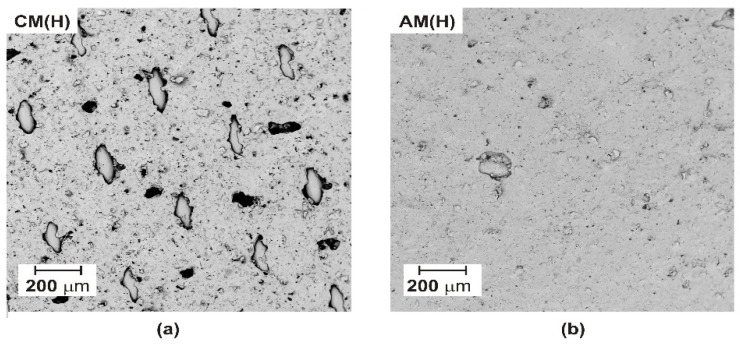
SEM images of the pristine ion-exchange membranes: (**a**) heterogeneous CM(H) membrane; (**b**) heterogeneous AM(H) membrane.

**Figure 2:**
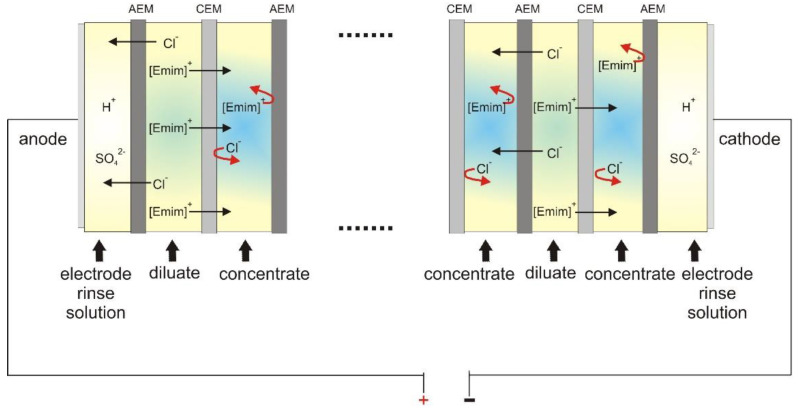
Scheme of the experimental ED module for [Emim]Cl recovery.

The ED experiments were carried out using an EDR-Z/10-0.8 module (MemBrain, Straz pod Ralskiem, Czech Republic) with an effective single-membrane area of 64 cm^2^. There were ten pairs of membranes in the ED stack. The ion-exchange membranes (IEMs) used in this investigation were heterogeneous AM(H)-CM(H) (Ralex, Straz pod Ralskiem, Czech Republic). The morphology of the tested heterogeneous ion-exchange membranes is shown in Figure 1. We proved that these membranes had a heterogeneous morphology, with the ion-exchange resin heterogeneously incorporated into the polymer membrane matrix. The anode and cathode in the electrodialyzer were made of platinized titanium. A scheme of the ED stack is shown in Figure 2.

The ED module consisted of the diluate, concentrate and electrode compartments. All ED experiments were conducted periodically with process solution recirculation. Process solutions such as the diluate, concentrate and electrode-rinse solution were recirculated by a peristaltic pump (Masterflex L/S, Cole-Parmer, Vernon Hills, IL, USA). The solution volumes were 200 mL for the diluate, 100 mL for the concentrate and 200 mL for the electrode rinse solution. The ED tests were conducted at constant voltage, which was a maximum value determined using the limiting current density test. The effects of the initial [Emim]Cl concentration (0.05 to 0.25 mol/L), different applied voltages (10, 15 and 20 V), different linear flow velocities (1 to 3 cm/s) and the diluate-to-concentrate volume ratio (2, 3, 4 and 5) were investigated. The experiments were performed until the diluate conductivity dropped to 0.25 mS/cm, which was monitored using a CPC-461 pH/conductivity meter (Elmetron, Zabrze, Poland). During the experiments, the electric current as well as the conductivities of the diluate and concentrate were measured every minute. The experiments were performed at 25 °C. For all of the ED experiments, three independent replicates were conducted, and the standard deviations were calculated.

Before the ED experiments, the surface morphology of the tested membranes was investigated using a scanning electron microscope (Hitachi TM3000 table-top TM series, Tokyo, Japan), equipped with a backscattered electron (BSE) detector.

### 2.3. Limiting Current Density (LCD)

The LCDs were determined based on current–voltage curves using the Cowan–Brown method [28], for solutions with [Emim]Cl concentrations of 0.05, 0.10, 0.15, 0.20 and 0.25 mol/L. The applied voltage was increased stepwise at a rate of 0.5 V/min until the ED cell potential drop reached 25 V.

### 2.4. Analytical Methods

**Figure 3:**
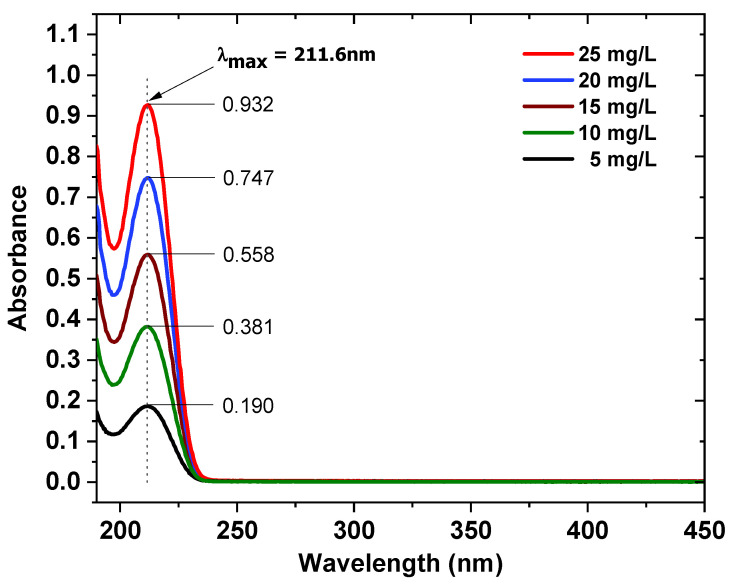
UV-VIS absorption spectra of [Emim]^+^ in [Emim]Cl solution at different [Emim]^+^ concentrations.

**Figure 4:**
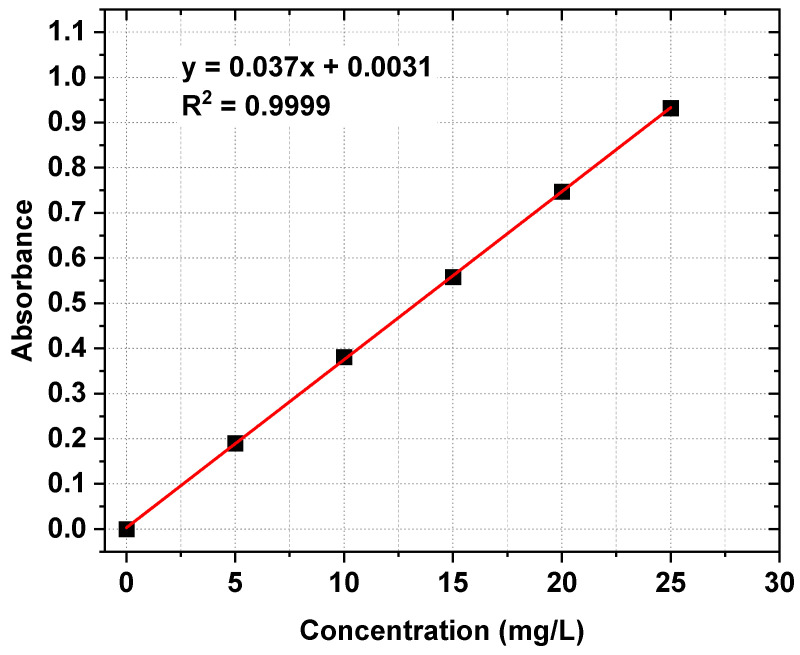
Calibration curve for measurement of [Emim]^+^ concentration.

The concentrations of the [Emim]Cl solutions were determined with a UV-VIS spectrophotometer (Varian Cary 50 Scan, Agilent, Santa Clara, CA, USA). The UV-VIS absorption spectra of [Emim]^+^ were measured in a 10 mm quartz cuvette. The spectrophotometer utilizes a dual-beam optical system, which can achieve spectral scanning over a wide wavelength range of 190 to 1100 nm. The UV-VIS spectra were pre-processed by baseline correction using deionized water as the reference. The maximum absorption wavelength for the [Emim]^+^ cation was 211.60 nm. The calibration curve method was applied. The linear correlation coefficient R^2^ for the obtained calibration curve was 0.9999. The [Emim]^+^ concentrations in experimental solutions were determined on the basis of the obtained calibration curve. Each analysis was performed in triplicate. The UV-VIS absorption spectra of [Emim]^+^ in [Emim]Cl solutions at different [Emim]^+^ concentrations and the calibration curve for measurement of the [Emim]^+^ concentration are shown in Figure 3 and Figure 4.

### 2.5. ED Effectiveness Determination

On the basis of the obtained results, the [Emim]Cl recovery ratio ($R_{[Emim]Cl}$), the [Emim]Cl concentration degree ($R_{conc}$), the electric current efficiency ($CE_{[Emim]Cl}$) and the energy consumption (*EC*) were calculated as follows:

- The [Emim]Cl recovery ratio ($R_{[Emim]Cl}$)

(1) $$R_{[Emim]Cl}=\frac{m_{IL,t}^{conc}}{m_{IL,0}^{dil}}\cdot 100\%$$

where $m_{IL,0}^{dil}$ is the initial mass (g) of the IL in the diluate solution before ED and $m_{IL,t}^{conc}$ is the increase in the IL mass (g) in the concentrate solution after ED.

- The [Emim]Cl concentration degree ($R_{conc}$)

(2) $$R_{conc}=\frac{C_{IL,t}^{conc_{}}}{C_{IL,0}^{dil_{}}}\cdot 100\%$$

where $C_{IL,0}^{dil}$ is the initial concentration (mol/L) of the IL in the diluate solution before ED and $C_{IL,t}^{conc_{}}$ is the final concentration (mol/L) of the IL in the concentrate solution after ED.

- The electric current efficiency ($CE_{[Emim]Cl}$)

(3) $$CE_{[Emim]Cl}=\frac{F\cdot z\cdot \frac{C_{IL,t}^{conc_{}}}{M_{IL}}\cdot V_{conc,t}}{n\cdot \int_{0}^{t}I(t)dt}\cdot 100\%$$

where *F* is the Faraday constant (96,485 C·mol^−1^), *z* is the charge number of [Emim]^+^, $V_{conc,t}^{}$ is the volume of the concentrate solution after ED (L), $C_{IL,t}^{conc_{}}$ is the concentration of the IL in the concentrate solution after ED (g·L^−1^), $M_{IL}$ is the molar mass of [Emim]Cl (g·mol^−1^), *n* is the number of membrane pairs and *I* is the electric current (A).

- Energy consumption (*EC*)

(4) $$EC=\frac{U\cdot \int_{0}^{t}I(t)dt}{V_{dil,0}}$$

where *EC* is the energy consumption, *U* is the applied voltage (V), *I* is the electric current in (A) and $V_{dil,0}^{}$ is the initial diluate volume (L).

## 3. Results and Discussion

The objective of the present study was to investigate the effectiveness of the [Emim]Cl recovery and concentration by following an optimized ED protocol. The effects of the initial [Emim]Cl concentration (0.05–0.25 mol/L), applied voltage (10–20 V), linear flow velocity (1–3 cm/s) and the diluate-to-concentrate volume ratio (2, 3, 4 and 5) are described in the following sections.

### 3.1. LCDs Determination

**Figure 5:**
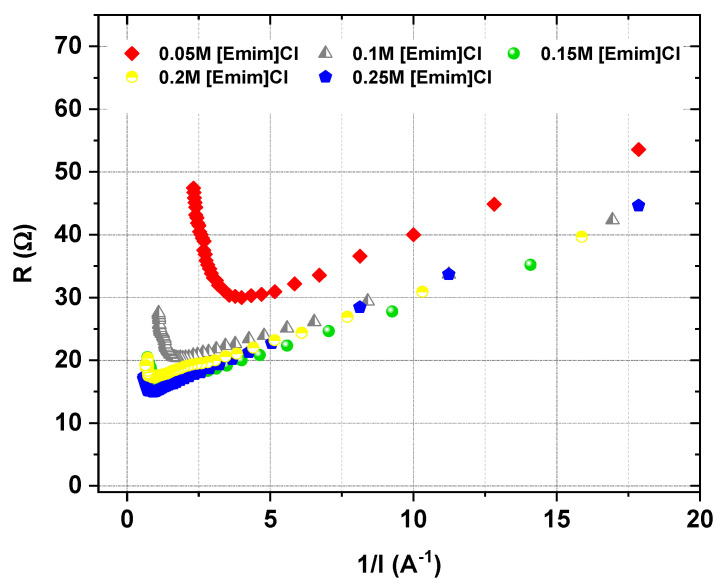
The dependence between the cell resistance and the current inverse.

In this study, firstly, the LCDs were determined. The LCDs of the [Emim]Cl solutions were determined using the relationship between the cell resistance and the reciprocal of the current (Cowan–Brown method [28]). As suspected, the LCD depended upon the [Emim]Cl concentration in the diluate solution. The obtained results are presented in Figure 5. We found that the LCD increased linearly with increasing [Emim]Cl concentration in the diluate. The determined LCD for the examined solutions 0.05 M, 0.1 M, 0.15 M, 0.2 M and 0.25 M [Emim]Cl equaled 39, 84, 135, 206 and 220 A/m^2^, respectively, which gave 7.5, 11, 14.5, 21 and 22 V, respectively. All of the ED desalination tests were carried out below the LCD at a constant voltage.

### 3.2. Effect of the Initial [Emim]Cl Concentration

The effect of the initial [Emim]Cl concentration on the effectiveness of the IL recovery and concentration was evaluated by testing five different starting concentrations of [Emim]Cl. The experimental conditions are presented in Table 1. The experiments were conducted in constant-voltage mode, but below the determined LCD, as previously mentioned. The initial diluate-to-concentrate volume ratio equaled 2.

**Figure 6:**
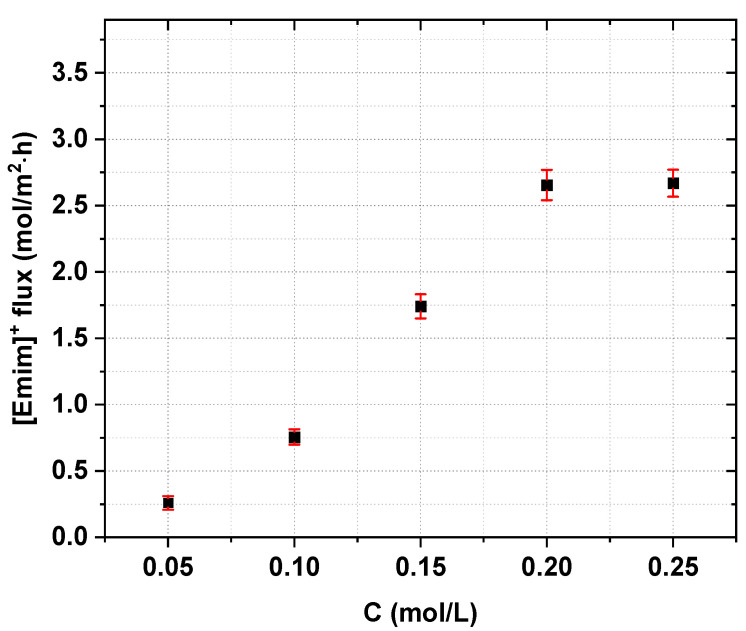
Influence of initial [Emim]Cl concentration on the [Emim]^+^ molar flux across cation-exchange membranes (*U* = 20 V, *w* = 2 cm/s).

**Figure 7:**
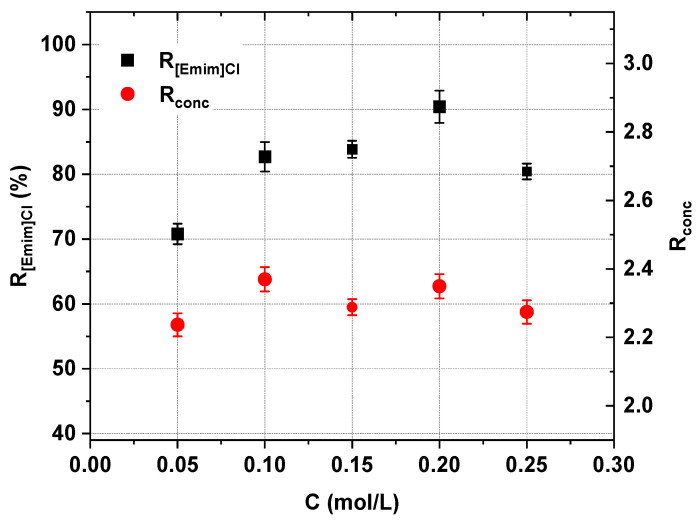
Influence of initial [Emim]Cl concentration on the [Emim]Cl recovery and concentration degree using the ED method (*U* = 20 V, *w* = 2 cm/s).

**Figure 8:**
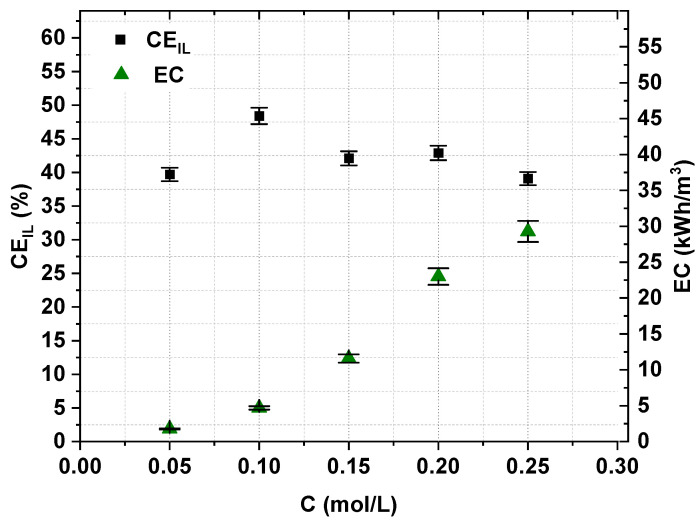
Influence of the initial [Emim]Cl concentration on ED current efficiency and energy consumption (*U* = 20 V, *w* = 2 cm/s).

The effect of the initial [Emim]Cl concentration on the performance of ED is presented in Figure 6, Figure 7 and Figure 8. We observed that the concentration of [Emim]Cl influenced the [Emim]^+^ flux across the cation-exchange membranes and the IL recovery (Figure 6 and Figure 7). The [Emim]^+^ flux and the [Emim]Cl recovery ratio increased with increasing [Emim]Cl concentration. When the initial [Emim]Cl concentration increased from 0.05 M to 0.2 M, the [Emim]^+^ flux increased from 0.26 to 2.66 mol/m^2^∙h, and the [Emim]Cl recovery increased from 70.8 to 90.4%. However, when the IL content in the initial diluate was raised to as high as 0.25 M, the [Emim]Cl recovery decreased to 80.4%, but the [Emim]^+^ flux did not change. However, within the margin of 0.05–0.2 M, it was also found that the initial IL concentration did not greatly influence the recovered IL concentration.

In all cases, the [Emim]Cl concentration degree was slightly higher than 2.2 (Figure 7). Moreover, as shown in Figure 8, the electric current efficiency did not depend strongly on the initial IL concentration. The electric current efficiency was in the range of 39–48%. The concentration of IL also impacted the membrane stack energy consumption. Figure 8 shows that with an increase in the initial IL concentration in the diluate, the energy consumption increased.

### 3.3. Effect of the Applied Voltage

**Figure 9:**
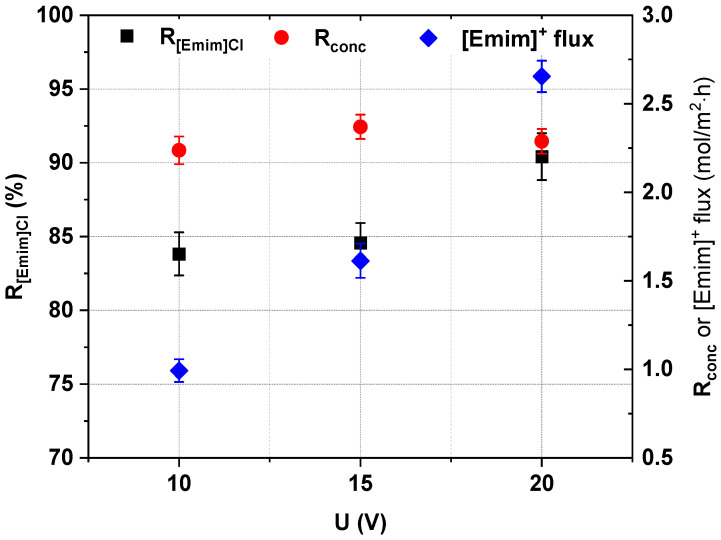
Influence of applied voltage on the [Emim]Cl recovery, concentration degree and [Emim]^+^ molar flux across cation-exchange membranes (0.2 M [Emim]Cl, *w* = 2 cm/s).

**Figure 10:**
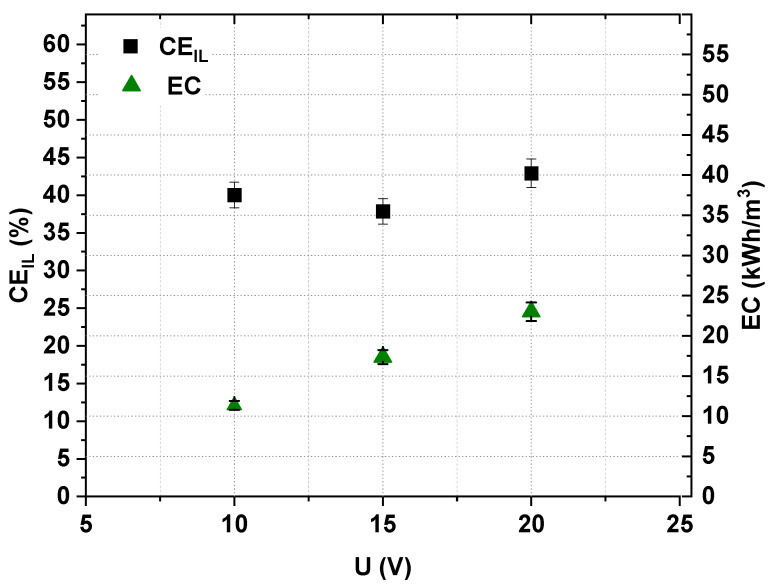
Influence of applied voltage on ED current efficiency and energy consumption (0.2 M [Emim]Cl, *w* = 2 cm/s).

The effect of the applied voltage on [Emim]Cl recovery and concentration effectiveness was evaluated by three separate ED experiments, which used a 0.2 M [Emim]Cl solution with a linear flow velocity equal to 2 cm/s. The examined voltages were 10 V, 15 V and 20 V. The experiments were conducted until the diluate’s conductivity was lower than 0.25 mS/cm. The ED effectiveness was evaluated according to the IL recovery ratio, the [Emim]Cl concentration degree, the [Emim]^+^ molar flux, the *CE* and the *EC*. The results are presented in Figure 9 and Figure 10.

We found that the applied voltage influenced the [Emim]Cl recovery and the [Emim]^+^ molar flux across cation-exchange membranes. The [Emim]Cl recovery and the [Emim]^+^ molar flux increased with an increase in the applied voltage, to maximum values of 90.42% and 2.66 mol/m^2^∙h, respectively, as shown in Figure 9.

Figure 9 shows that the [Emim]Cl concentration degree also depended on the applied voltage during ED. The [Emim]Cl concentration degree increased slightly with an increase in the applied voltage. The maximum value of the [Emim]Cl concentration degree was 2.35 for 15 V. Moreover, while the applied voltage increased from 10 V to 15 V, the current efficiency slightly decreased to values near 38% (Figure 10). However, when the voltage increased from 15 V to 20 V, the current efficiency slightly increased to 42.9%. As shown in Figure 10, the energy consumption increased with increasing applied voltage.

### 3.4. Effect of the Linear Flow Velocity

**Figure 11:**
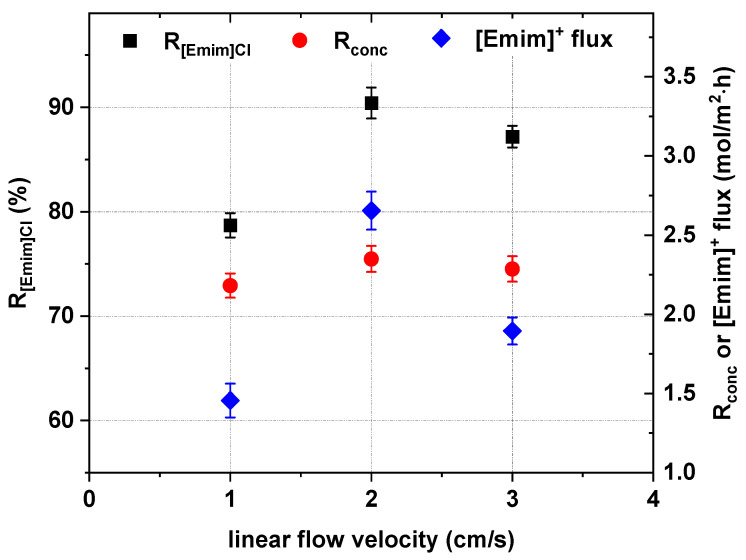
Influence of linear flow velocity on the [Emim]Cl recovery, concentration degree and [Emim]^+^ molar flux across cation-exchange membranes (0.2 M [Emim]Cl, *U* = 20 V).

**Figure 12:**
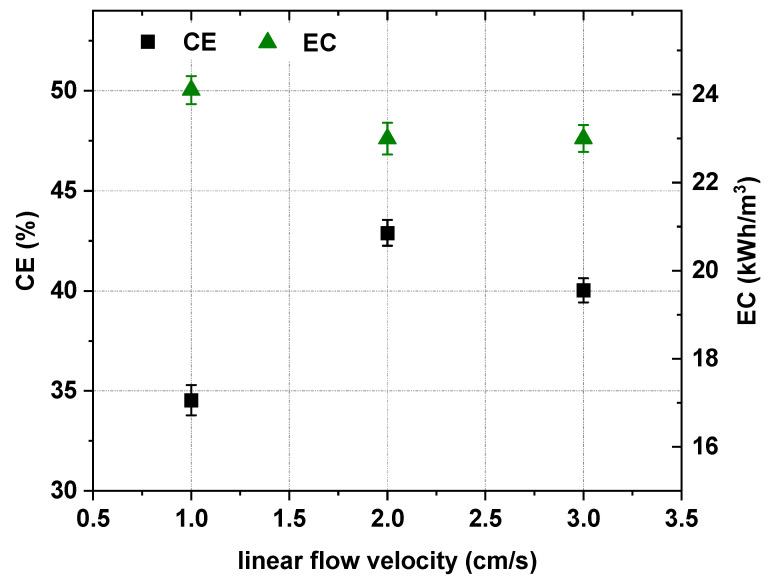
Influence of linear flow velocity on ED current efficiency and energy consumption (0.2 M [Emim]Cl, *U* = 20 V).

One of the important parameters which influences ED efficiency is fluid dynamics. In this section, the effect of the linear flow velocity on the effectiveness of [Emim]Cl concentration and recovery was evaluated by three simultaneous ED experiments conducted using the same [Emim]Cl concentration (0.2 M) and voltage drop (20 V). The examined linear flow velocities were 1 cm/s, 2 cm/s and 3 cm/s. The effect of the linear flow velocity on ED effectiveness is shown in Figure 11 and Figure 12. The linear flow velocity slightly influenced the energy consumption. When the solution flow velocity increased from 1 cm/s to 2 cm/s and 3 cm/s, the energy consumption decreased from 24.1 kWh/m^3^ to 23 kWh/m^3^ (Figure 12). The linear flow velocity, and consequently the residence time of ions inside the electrodialyzer compartments, influenced the [Emim]^+^ molar flux, the [Emim]Cl recovery ratio, the [Emim]Cl concentration degree and the electric current efficiency. When the solution flow velocity increased from 1 cm/s to 2 cm/s, the [Emim]^+^ molar flux across cation-exchange membranes dramatically increased to a maximum value of 2.66 mol/m^2^∙h. Therefore, the flow velocity had a significant impact on the effectiveness of ED in this context. The best ED efficiency was obtained when the linear flow velocity was 2 cm/s. When the applied solution flow velocity was 2 cm/s, the [Emim]Cl recovery, concentration degree and current efficiency increased to the maximum values of 90.4%, 2.35 and 42.9%, respectively, as shown in Figure 11 and Figure 12.

### 3.5. Effect of the Diluate-to-Concentrate Volume Ratio

**Figure 13:**
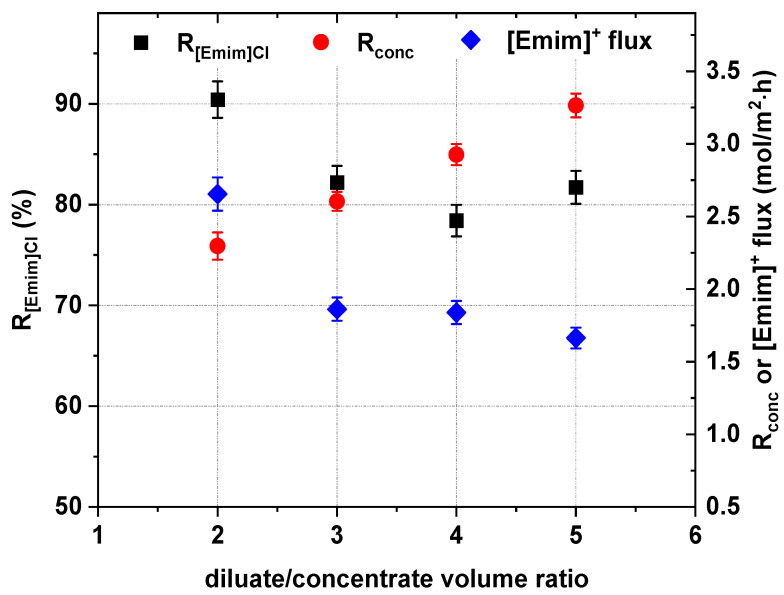
Influence of the diluate-to-concentrate volume ratio on the [Emim]Cl recovery, concentration degree and [Emim]^+^ molar flux across cation-exchange membranes (0.2 M [Emim]Cl, *U* = 20 V, *w* = 2 cm/s).

**Figure 14:**
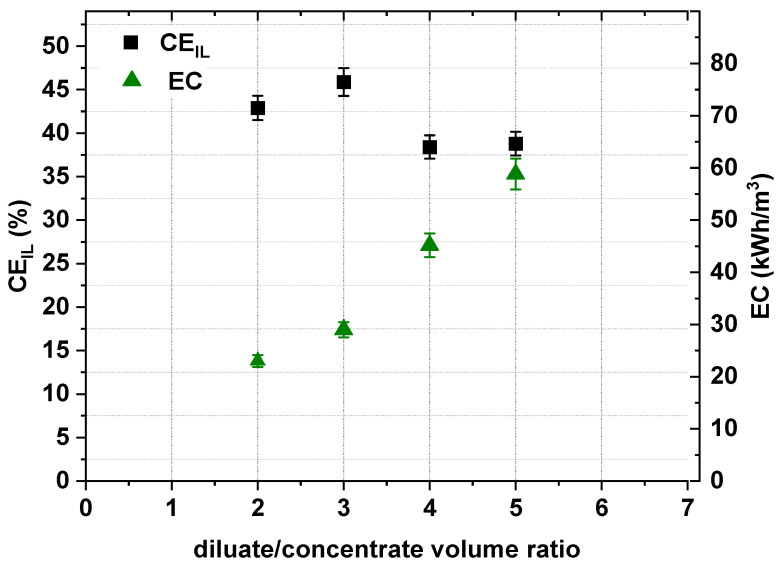
Influence of diluate-to-concentrate volume ratio on ED current efficiency and energy consumption (0.2 M [Emim]Cl, *U* = 20 V, *w* = 2 cm/s).

The IL concentration degree depends on the initial diluate volume. When the diluate-to-concentrate volume ratio is high, the concentration degree should be greater in comparison with a low ratio. However, the trade-off in having a high concentration degree is that the concentration difference between the diluate and concentrate during ED is higher [1,29]. This promotes electro-osmotic water transport and the concentration gradient between the diluate and concentrate. Figure 13 shows that, as suspected, the [Emim]Cl concentration degree increased with increasing initial diluate volume. When the diluate-to-concentrate volume ratio was 5, the concentration degree increased to a maximum value of 3.35 (in the examined range). Additionally, the energy consumption increased with the increase in the initial diluate volume (Figure 14). However, while the initial diluate volume increased, the concentration gradient between diluate and concentrate increased, and the [Emim]^+^ molar flux across cation-exchange membranes dramatically decreased (Figure 13). Thus, it was noted that when the diluate-to-concentrate volume ratio increased from 2 to 5, the [Emim]Cl recovery and current efficiency decreased from 90.4% to 81.7%, and from 42.9% to 38.8%, respectively (Figure 13 and Figure 14).

### 3.6. Comparison of ILs Recovery Performance Using the ED Method

Because ILs are gaining important roles in various industrial fields, their recovery is also becoming important. Table 2 presents a comparison of the recovery effectiveness of commonly used ILs using the ED method.

The results presented herein confirm that the ED operating parameters and the initial IL concentration in the diluate strongly influenced the recovery effectiveness of ED. The observed [Emim]Cl recovery ratios presented in this work were high compared to the data provided in the literature for [Amim]Cl, [Bmim]Cl and [Bmim]Br [14,15,23,24]. It was proven that ED allowed up to 90.4% [Emim]Cl recovery at 20 V at the initial IL concentration of 0.2 M. The lower [Bmim]Cl and [Bmim]Br recovery coefficient in comparison to [Emim]Cl recovery can be explained by the difference in the cation size influencing the ion transport across ion-exchange membranes. The recovery of ILs also depends on the IL concentration in the diluate. Table 2 shows that the ILs recovery coefficient for 0.25 M [Amim]Cl [14] was lower compared to the ILs recovery obtained for 0.2 M [Emim]Cl, which has a smaller ionic radius. The ILs content in the diluate also influenced the ILs transport rate. The ILs transport rate increased with increasing IL concentration in the initial diluate solution. The current efficiency also highly depended on ED performance. The applied voltage also influenced ED performance. When the voltage increased, the current efficiency slightly increased [15]. Table 2 shows that IL recovery above 90% can be also obtained by the application of electrodialysis with a bipolar membrane (BMED) [25,27]. BMED is a method for the generation of H^+^ and OH^–^ ions, which can be used to produce bases and acids from salts [30]. The results presented in Table 2 indicate that it is very important to select the optimal ED process operating parameters.

## 4. Conclusions

The various industries that use ILs generate a wide range of wastewaters containing dilute ILs. Because this type of waste could have a negative effect on the environment and human health by polluting the water, air and soil, the recovery of ILs constitutes a very important initiative.

In the current study, the possibility of simultaneous [Emim]Cl recovery and concentration from aqueous solutions by ED was investigated. It was observed that [Emim]Cl was removed from the diluate effectively using the proposed method. The obtained results proved that a recovery of up to 90.4% is possible. It was also proven that the ED process could be optimized to allow for concentration of the IL. In fact, the [Emim]Cl content in the examined process increased by 2.55 times in comparison to the IL concentration in the initial diluate compartment. At a diluate-to-concentrate volume ratio of 5, a 3.35-fold concentration of [Emim]Cl, with a recovery of 81.7%, could be obtained. It was shown that the IL concentration, applied voltage drop, linear flow velocity and diluate-to-concentrate volume ratio all had an influence on the recovery efficiency and concentration of the chosen IL. The highest ED efficiency for the recovery and concentration of [Emim]Cl was obtained at 20 V applied potential, 2 cm/s linear flow velocity and 0.2 M IL in the feed solution.

The performed experiments allowed the operating parameters of [Emim]Cl recovery by ED to be optimized; therefore, the obtained results could be a valuable base to develop and scale-up the method for the recovery and concentration of [Emim]Cl from industrial wastewater. In future, the ED methodology presented here could be a good way to recycle ILs and be a highly valuable wastewater utilization method, especially for wastewater originating from biomass utilization processes or the pharmaceutical industry.

## Figures and Tables

**Table 1:** The experimental conditions.

Exp. No.	Initial Diluate	Initial Concentrate	Applied Voltage, V
1.	200 mL of 0.05 M [Emim]Cl	100 mL of 0.05 M [Emim]Cl	7
2.	200 mL of 0.1 M [Emim]Cl	100 mL of 0.1 M [Emim]Cl	10
3.	200 mL of 0.15 M [Emim]Cl	100 mL of 0.15 M [Emim]Cl	14
4.	200 mL of 0.2 M [Emim]Cl	100 mL of 0.2 M [Emim]Cl	20
5.	200 mL of 0.25 M [Emim]Cl	100 mL of 0.25 M [Emim]Cl	20

**Table 2:** Comparison of the selected ILs’ recovery performance using the ED method.

Initial Diluate Composition	Process	Voltage, V	Flow Rate, L/min	Linear Flow Velocity, cm/s	Diluate-to-Concentrate Volume Ratio	ILs Recovery, %	Transport Rate, mol/h·m^2^	Current Efficiency, %	Ref.
0.2 M [Emim]Cl	ED	20	0.18	2	2:1	90.4	2.67	42.9	this work
0.25 M [Amim]Cl	ED	15	5	-	1:1	66–68	2.0–3.1	62–68	[14]
0.18 M [Bmim]Br	ED	20	3	-	1:1	60.3	-	63.4	[23]
0.01 M [Bmim]Cl	ED	10	1.56	-	1:1	57.4	0.23	37.7	[15]
0.04 M [Bmim]Cl	ED	10	1.56	-	1:1	74.1	0.46	70.7	[15]
0.04 M [Bmim]Cl	ED	3	1.56	-	1:1	59.7	0.46	60.6	[15]
0.3 M [Bmim]Cl	ED	15	0.5	-	1:1	49	-	30	[24]
0.3 M [Bmim]Cl	ED	20	0.5	-	1:1	70	-	55	[24]
0.2 M [Bmim]HSO_4_	BMED	-	20	-	1:1	94	5.07	89	[25]
0.2 M [TEA]HSO_4_	BMED	-	25	-	1:1	90	4.2	88	[27]

## Data Availability

The data presented in this study are available on request from the corresponding author.
